# The Importance of Nutrition in Hypertension

**DOI:** 10.3390/nu11102542

**Published:** 2019-10-21

**Authors:** Francesco Fantin, Federica Macchi, Anna Giani, Luisa Bissoli

**Affiliations:** Department of Medicine, Section of Geriatrics, University of Verona, Healthy Aging Center, Piazzale Stefani 1, 37126 Verona, Italy; federica.macchi92@gmail.com (F.M.); annagiani92@gmail.com (A.G.); luisa.bissoli@aovr.veneto.it (L.B.)

Arterial hypertension (AH) is considered to be one of the most relevant cardiovascular risk factors, and its wide prevalence in all age ranges makes it necessary to analyse all the possible causes and treatments. In this special issue, nutritional interventions are examined either as causes or as treatments of AH.

Several studies have been considered in the five reviews and three communications, which, along with six articles, compose the current issue. 

Five articles [1,2,3,4,5] and one review [6] explain the possible blood pressure (BP) lowering effects of different nutritional elements. 

In animal models, hesperidin [2], a flavanone glycoside contained in citrus fruits, has been shown to reduce blood pressure, left ventricular hypertrophy and cardiac fibrosis by the down regulation of transforming growth factor-beta 1 (TGF-beta1) and tumor necrosis factor-receptor 1 (TNF-R1) expression, as well as the reduction of TGF-beta1 plasma levels. Furthermore [4], Ojeoksan, which is a mixture of 17 herbal medicines, first described in ancient Korean medicinal literature, has been shown to improve vascular function and significantly reduce inflammatory processes, giving positive results both on vascular relaxation and on atherosclerosis prevention. 

In an animal model [3], vasorelaxation, and consequently blood pressure reduction, can also be obtained by short-period administration of protocatechuic acid (PCA), a natural phenolic compound found in many types of food, as described in an interesting study by Kunanya. Moreover, this study demonstrates the strong anti-oxidant effects of PCA on aging hypertension. 

On the other hand, beetroot juice intake should be carefully considered; even if it increases the nitric oxide (NO) salivary concentration in post-menopausal women, it does not show significant effects on BP control. In a study on 13 hypertensive post-menopausal women [1] undergoing beetroot juice administration and moderate-intensive aerobic exercise, no significant BP reduction was observed. The possible effects of high intake of fish are analysed in Vildmyren’s study [5]; a press-cake meal (water-insoluble proteins obtained from cod residual materials), given to obese Zucker fa/fa rats, was found to prevent or delay high blood pressure through inhibition of renin-angiotensin-system (RAAS).

An exhaustive review by Li et al. evaluates the positive anti-hypertensive effects of tea and tea-metabolites, confirming that both green and black tea may reduce BP. Actually, not all of the studies included in this wide review lead to positive outcomes. Potential confounding elements should be carefully considered, such as the duration of tea consumption, the origin of tea and mainly the transient short-term increase in BP determined by caffeine, which is contained in tea. Further studies are needed to better describe the molecular mechanism underlaying tea effects on oxidative stress, vascular relaxation and inflammation.

On the other hand, [7] fructose assumption, which has become common globally, is responsible for BP increase, acting both on renal sodium reabsorption and on the sympathetic nervous system (SNS). 

A double effect is associated with aminoacids and electrolytes, which is shown in two communications and in a review [8,9,10]. 

Plasma or urinary aminoacids concentrations [8] were studied in order to determine an association with BP level; for example, phenylalanine shows a positive relation with systolic and diastolic BP, whereas glutamic acid seems to lower systolic and diastolic BP. A considerable number of studies are included in this review and the heterogeneity of study results analysed in this review does not allow unique conclusion to be drawn. 

Sodium, potassium, calcium and magnesium may have different impacts on BP levels [9] and the review of several meta-analyses confirms the well-known beneficial effect of low sodium and increased potassium intake. On the other hand, regarding magnesium intake, just moderate results were achieved [9]. 

Calcium plasma level [10] is noteworthy: an increase in calcium assumption is found to lower PB levels, both by parathyroid hormon (PTH)-signalling and by renin angiotensin aldosteron system (RAAS) pathway regulation; a major effect was found in subjects with baseline low calcium intake.

It has been widely shown that sodium intake is strictly related to an increase in blood pressure levels. As explained in the review by Grillo et al. [11], several mechanisms, such as water retention, increase in systemic peripheral resistance, endothelial dysfunction with changes in the structure and function of large elastic arteries, together with modification in sympathetic activity and in the autonomic neuronal modulation of the cardiovascular system, are involved in the relationships between high salt intake and risk of hypertension.

The importance of nutritional intervention is also crucial in pregnancy; in fact, as demonstrated in animal models [12], the unbalanced maternal nutrition has a relevant impact on foetal programming leading to programmed hypertension. 

An interesting observational study [13] conducted on a large cohort of primary school children demonstrates that high BP and obesity are strongly linked to unhealthy dietary patterns; these subjects also presented impaired pulse wave-velocity and capillary cholesterol. Therefore, lifestyle interventions and a nutritionally balanced diet, such as the Mediterranean diet [14], are highly recommended in all subjects and, in particular, among obese people. In line with this, in our review [15], we focused on obese subjects and we underlined the huge effect of life-style modification intervention on BP management. Moreover, the positive effects of bariatric surgery and pharmacological intervention are also considered, with an aim to reduce body weight and BP at the same time. 

Therefore, in our opinion, the encouraging findings gathered in this special issue provide evidence for further research and considerations. Firstly, the interesting results achieved with animal models should be confirmed in the human population. Secondly, we think that this special issue confirms that BP level control should start from a healthy nutritionally balanced diet, which should be pursued all through life, and even before birth.
